# Nationwide survey on the management of pediatric pharyngitis in Italian emergency units

**DOI:** 10.1186/s13052-023-01514-8

**Published:** 2023-09-05

**Authors:** Gregorio P. Milani, Claudio Rosa, Naz Tuzger, Ilaria Alberti, Chiara Ghizzi, Stefania Zampogna, Angela Amigoni, Carlo Agostoni, Diego Peroni, Paola Marchisio, Elena Chiappini, IPSE study group, Eleonora Tappi, Ivana Rabbone, Filippo M. Salvini, Giorgio Cozzi, Davide Silvagni, Marco Pitea, Sergio Manieri, Antonella Crisalfi, Angelina Vaccaro, Anna M. Plebani, Susanna Falorni, Laura Martelli, Marcello Lanari, Giuseppe Di Stefano, Mauro De Martinis, Pasquale Bulciolu, Lorenzo Iughetti, Elisabetta Fabiani, Rita Greco, Fabio Cardinale, Riccardo Boera, Alberto Arrighini, Antonio Chiaretti, Felice Nunziata, Rosario Salvo, Giuseppe Banderali, Silvia Fasoli, Gabriella Baracchia, Roberto Antonucci, Andrea Biondi, Andrea Tenci, Gianpaolo Mirri, Laura Battisti, Massimo Bellettato, Ermanno Ruffini, Paola Cogo, Danica Dragovic, Liviana Da Dalt, Paola Bruni, Mimma Caloiero, Tiziana Varisco, Marcello Palmieri, Emanuela Picciotti, Antonio Cualbu, Ugo Pradal, Salvatore Grosso, Laura Maria Pogliani, Angelo Selicorni, Massimo Soffiati, Pietra Bene, Andrea Guala, Giuseppe Bertolozzi, Paola Tommasi, Angelo Campanozzi, Andrea Cella, Cesare Ghitti, Paolo Groff, Claudia Bondone, Antonio Vitale, Pina Teresa Capalbo, Roberto Dall’Amico, Antonio Sisto, Ecclesio L. Livio, Emanuele Tatò, Marina Flora, Giuseppe Raiola, Agnese Suppiej, Ciro Clemente, Francesca Lizzoli, Francesco Chiarelli, Alberto Podestà, Fabio R. Forte, Pierluigi Vasarri, Guido Pennoni, Flavia Parrinello, Martina Fornaro, Roberto della Casa, Claudia Bondone, Federico Zaglia, Vincenzo Tipo, Francesco Morandi, Valerio Cecinati, Giuseppe Calabrò, Beatrice Messini, Gian Luigi Marseglia, Sergio Arrigoni, Santina Gaggiano, Claudio Cavalli, Giuseppe Gramaglia, Laura Serra, Enrico Valletta, Gaia Militerno, Massimo Chiossi, Gino Camellino, Stefano Masi, Federico Marchetti, Giovanni Traina, Rosa Maria Maccarrone

**Affiliations:** 1 Pediatric Unit, Foundation IRCCS Ca’ Granda, Ospedale Maggiore Policlinico, Via Della Commenda 9, 20122 Milan, Italy; 2 Department of Clinical Sciences and Community Health, Università Degli Studi Di Milano, Milan, Italy; 3 AUSL Bologna, Ospedale Maggiore, Bologna, Italy; 4 SOC Pediatria, Azienda Sanitaria Provinciale Di Crotone, Crotone, Italy; 5 Pediatric Intensive Care Unit, University Hospital of Padua, Padua, Italy; 6 Department of Clinical and Experimental Medicine, Section of Pediatrics, University of Pisa, Pisa, Italy; 7 Department of Pathophysiology and Transplantation, University of Milan, Milan, Italy; 8 Pediatric Infectious Disease Unit, Department of Health Sciences, Meyer Children’s University Hospital, University of Florence, Florence, Italy

**Keywords:** Group A β-hemolytic streptococcus. Management, Antibiotic stewardship, Over prescription, Test

## Abstract

**Background:**

Acute pharyngitis is a frequent reason for primary care or emergency unit visits in children. Most available data on pharyngitis management come from primary care studies that demonstrate an underuse of microbiological tests, a tendency to over-prescribe antibiotics and a risk of antimicrobial resistance increase. However, a comprehensive understanding of acute pharyngitis management in emergency units is lacking. This study aimed to investigate the frequency of rapid antigen test use to diagnose acute pharyngitis, as well as other diagnostic approaches, the therapeutic attitude, and follow-up of children with this condition in the emergency units.

**Methods:**

A multicentric national study was conducted in Italian emergency departments between April and June 2022.

**Results:**

A total of 107 out of 131 invited units (response rate 82%), participated in the survey. The results showed that half of the units use a scoring system to diagnose pharyngitis, with the McIsaac score being the most commonly used. Most emergency units (56%) were not provided with a rapid antigen diagnostic test by their hospital, but the test was more frequently available in units visiting more than 10,000 children yearly (57% vs 33%, respectively, p = 0.02). Almost half (47%) of the units prescribe antibiotics in children with pharyngitis despite the lack of microbiologically confirmed cases of Group A β-hemolytic streptococcus. Finally, about 25% of units prescribe amoxicillin-clavulanic acid to treat Group A β-hemolytic streptococcus pharyngitis.

**Conclusions:**

The study sheds light on the approach to pharyngitis in emergency units, providing valuable information to improve the appropriate management of acute pharyngitis in this setting. The routinary provision of rapid antigen tests in the hospitals could enhance the diagnostic and therapeutic approach to pharyngitis.

**Supplementary Information:**

The online version contains supplementary material available at 10.1186/s13052-023-01514-8.

## Introduction

Acute pharyngitis is one of the most common infections and reasons for primary care or emergency unit visits in childhood [1, 2]. Streptococci typically account for approximately one third of acute pharyngitis cases, with the majority being secondary to viral pathogens [3].

Since it is not possible to clinically distinguish Group A β-hemolytic streptococcus from viral pharyngitis, most international guidelines advise a combination of scoring systems and microbiological testing to facilitate its correct diagnosis and treatment [4]. Despite these recommendations, inappropriate management of pharyngitis is a worldwide issue that leads to its under or overtreatment [5]. This phenomenon not only impact the disease course but also contribute to the rise in antimicrobial resistance [6].

The available data on the management of pharyngitis in clinical practice mainly come from primary care studies, which demonstrate an underuse of microbiological tests and a tendency to over-prescribe antibiotics [7]. However, a comprehensive understanding on acute pharyngitis management in the emergency units is lacking. Therefore, we conducted a nationwide study to shed light on the approach to pharyngitis cases in the emergency unit. The primary aim of the study was to investigate the frequency of rapid antigen test use to diagnose acute pharyngitis. The secondary aims included the assessment of other diagnostic approach, the therapeutic attitude and follow-up of children with this condition.

## Materials and methods

In this multicentric national study, we created and distributed the Italian-Pharyngitis-Survey-in Emergency (IPSE) among Italian emergency departments (both general and pediatric) between April-June 2022. Directors of the emergency units were invited by email to participate in this survey facilitated via the Google Forms platform. To obtain a comprehensive national data set, all directors of emergency units affiliated to the two national scientific society of pediatric emergency care (AMIETIP “Accademia Medica Infermieristica di Emergenza e Terapia Intensiva Pediatrica” and SIMEUP “Società Italiana di Emrgenza Urgenza Pediatrica”) were invited. Furthermore, we tentatively invite at least one director in each Italian province [8].

One email reminder was sent every two weeks for a total of three reminders to non-respondents. If there was no answer, the emergency unit was tentatively contacted by phone.

### Questionnaire development

Three international experts of pediatric infectious diseases (P.M., E.C. and D.P.) together with two experts of pediatric emergency medicine (G.P.M. and M.G.B) created the survey based on the international guidelines on pharyngitis management. A pilot test was run among 10 emergency physicians and modified based on their comments. Then, two further emergency physicians answer the full questionnaire for two times after a 14-day interval. The intra-rater reproducibility turned out to be 96%. The final version of the questionnaire (Supplementary material) was made up of three main sections: 1) The first section addressed the characteristics of the emergency department including the name and place of the hospital; the total number of children visited yearly in the emergency unit and the number of children with pharyngitis; and the availability of written recommendations on pharyngitis diagnosis, treatment, and follow-up in the emergency unit. 2) The second section investigated the use of scoring systems, rapid antigen tests or throat culture to diagnose pharyngitis. 3) The third section addressed the choice of first and second-line antimicrobial treatment for pharyngitis (both for isolated or recurrent cases), the frequency of consultation of sub-specialists (otorhinolaryngologist or infectious diseases specialists), and the follow-up of ascertained cases. Most questions were structured with only a single choice possible.

### Data analysis

Categorical variables were reported as frequencies and percentages. To investigate possible relation between the number of children (total and with acute pharyngitis) visited per year and the use of rapid antigen tests, the Fisher exact test was employed. A *p* < 0.05 was assumed as significant. As it was mandatory to fill out the entire survey, no missing data was expected.

## Results

### Participant characteristics

A total of 107 out of 131 invited units (response rate 82%), participated in the survey. The full list of participating units is provided within the online Supplementary material. Table 1 shows the characteristics of the Italian emergency units involved in the survey. Approximately half of the units (*N* = 54, 50%) disclosed > 10,000 visits and > 500 pharyngitis cases yearly. Most units have written internal guidelines (*N* = 78, 73%).

**Table 1:** Characteristics of the emergency units participating the survey

Number of visits per year	N (%)
<5000	28 (26)
5000–10,000	26 (24)
10,000–20,000	41 (38)
>20,000	12 (11)
Number of pediatric pharyngitis managed per year	
<100	9 (8.4)
100-500	42 (39)
500-1000	29 (27)
>1000	27 (25)
Availability of written recommendations on pharyngitis management	
Yes	78 (73)
No	29 (27)

### Diagnostic approach to pediatric pharyngitis cases

#### Use of scoring systems

**Fig. 1:**
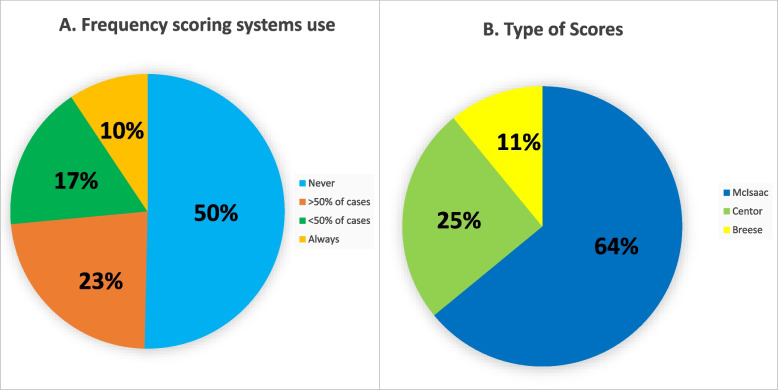
(left panel): Frequency of the use of clinical scoring systems in the Italian emergency units**; **(right panel): Type of clinical score systems utilized in the Italian emergency units

A scoring system is utilized to diagnose pharyngitis in half of the units (*N* = 53, 50%). Most units use the McIsaac score (*N* = 42, 81%) and the remaining units utilize either the Centor (*N* = 6, 12%) or the Breese Score (*N* = 4, 7.7%) as showed in Fig. 1.

#### Rapid antigen diagnostic test

Most emergency units (*N* = 60, 56%) cannot rely on a rapid antigen diagnostic test supplied by their hospital. The test was more frequently available in units visiting > 10.000 children yearly as compared to the other units (57% vs 33%, respectively, *p* = 0.02). No difference was found considering units managing > 500 pharyngitis yearly as compared with other units (49% vs 40%, respectively, *p* = 0.44).

Among units provided with the antigen rapid test, ~ 70% use the test at the emergency physician, ~ 20% utilize it based on scoring systems and the remaining 10%) use it routinary in all children with acute pharyngitis (Fig. 1).

To perform the rapid antigen test, the posterior pharyngeal wall and the palatine tonsils are scraped by most units (*N* = 28, 60%). The palatine tonsils only (*N* = 12, 26%), the oral cavity, posterior pharyngeal wall, and the palatine tonsils (*N* = 4, 8.5%) and the posterior pharyngeal wall only (*N* = 3, 6.4%) are scraped by the remaining units.

#### Throat culture

About 40% of units perform throat cultures as an alternative diagnostic test to rapid antigen test (*N* = 45, 42%). The use of a throat culture is based on the discretion of the individual doctor in almost 90% of units (*N* = 39, 89%).

### Therapeutic approach

#### Antimicrobial therapy

An antimicrobial therapy is prescribed based on a clinical suspicion (clinical history and physical examination) for Group A β-hemolytic streptococcus in 47% of units (*N* = 50) whereas it is prescribed based on a positive rapid antigen test in 38% of units (*N* = 41). A minority of units (*N* = 16, 15%) prescribe antimicrobial treatment only after a positive throat culture.

Three out of four units (*N* = 81, 76%) prescribe Amoxicillin as antibiotics of first choice, whereas the remaining (*N* = 26, 24%) make use of Amoxicillin-Clavulanic Acid. For these two antibiotics, 50 mg/kg/day is the posology chosen by 71% of units. Only 2% of units prescribe them at a dose lower than 50 mg/kg/day. The remaining 27% of units prescribe Amoxicillin or Amoxicillin-Clavulanic Acid at a dose > 50 mg/kg/day. A 10-day regimen is prescribed by 45% of units, whereas 53% prescribe a shorter treatment.

In the case of recurrent pharyngitis from Group A β-hemolytic streptococcus, the first choice of antibiotics in most units (*N* = 58, 54%) is Amoxicillin-Clavulanic Acid. Amoxicillin is prescribed by 26% of units, cephalosporins by 15% and macrolides by ~ 5%.

#### Follow-up

**Fig. 2:**
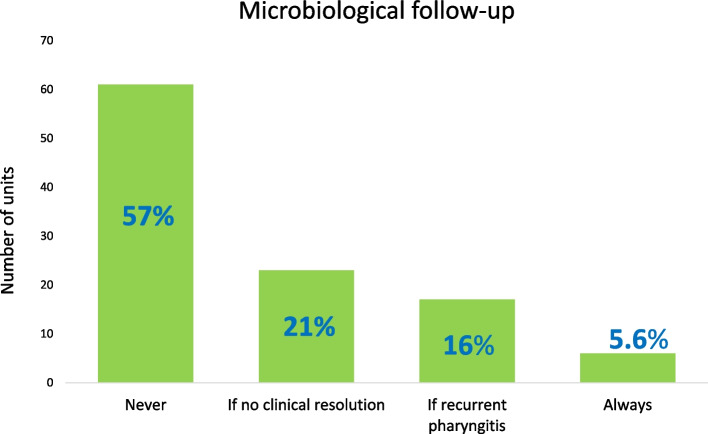
Use of microbiological follow-up in children with Group A β-hemolytic streptococcus

A microbiological follow up with rapid antigen test or a throat culture is never performed in 57% (*N* = 61) of units. A follow-up is prescribed by 20% (*N* = 23) of units if there is no symptom resolution and 16% (*N* = 17) only in case of recurrent pharyngitis A minority of units (*N* = 6, 5.6%) always follow up patients with a secondary diagnostic test (Fig. 2).

#### Specialists’ consultation

An otorhinolaryngologist specialist is consulted in case of local suppurative complications in the majority (*N* = 99, 93%) of units. In a low percentage of units, this specialist is consulted in case of fever that does not resolve after 3-day antimicrobial therapy (*N* = 5, 4.7%). In the remaining units (*N* = 6, 5.6%), this specialist is never consulted.

Most units never consult an infectious disease specialist (*N* = 70, 65%). The main reasons to call an infectious diseases specialist is the presence of fever for more than 3 days in course of antimicrobial treatment and local complications (*N* = 23, 21% and *N* = 15, 14% respectively). A small percentage does not have an infectious diseases specialist in their hospital (*N* = 4, 3.7%).

The remaining results of the survey are reported within the online Supplementary material.

## Discussion

This nationwide survey revealed that only a minority of Italian emergency units utilize both scoring systems and rapid antigen test to manage pediatric patients with pharyngitis. Moreover, despite the estimated prevalence of streptococcal group A pharyngitis being ~ 25% among Italian children [9], a noteworthy proportion of pediatric patients are administered antibiotics without confirmed microbiological evidence of Group A β-hemolytic streptococcus infection. Lastly, one in four units administer amoxicillin-clavulanate as the primary treatment for Group A β-hemolytic streptococcus pharyngitis. The discussion will focus on the following aspects 1) the role rapid antigen test in the emergency units 2) the therapeutic approach of Group A β-hemolytic streptococcus and 3) The implications of this survey data for antimicrobial resistance.

The findings of this study indicate that rapid antigen testing is accessible in less than 50% of the emergency units in Italy and is primarily utilized independently of clinical scores. Moreover, it reveals that the availability of this test is lower in units that attend to less than 10,000 patients annually compared to those that see higher patient volumes. A Cochrane review published in 2020 demonstrated that the implementation of rapid antigen testing is correlated with a 25% decrease in antibiotic prescriptions for pharyngitis [10]. Furthermore, a previous Cochrane limited to pediatric populations found that the sensitivity and specificity of rapid antigen testing for the detection of Group A β-hemolytic streptococcus pharyngitis are greater than 85% [11]. On the other hand, a study conducted in Brazil found that utilizing rapid antigen testing in the emergency unit resulted in a 40% rise in the detection of Group A β-hemolytic streptococcus infection in children with pharyngitis compared to relying solely on clinical assessment [12]. The findings of our survey and existing evidence on the efficacy of rapid antigen testing underscore the significance of ensuring that emergency units are equipped with rapid antigen tests by healthcare systems. Future multicenter international study should investigate if such tests are routinely provided in emergency units of other countries.

Regarding the therapeutic approach, nearly 25% of Italian emergency units administer amoxicillin-clavulanate as the initial course of treatment for Group A β-hemolytic streptococcus pharyngitis. Both amoxicillin and penicillin have proven efficacy in treating this bacterial infection [13, 14]. Therefore, the utilization of antibiotics with a wider spectrum of activity does not confer any benefits and is linked to a heightened risk of adverse effects [15]. These side effects are primarily attributable to the modification of the intestinal microbiota caused by amoxicillin-clavulanate, resulting in diarrhea [16]. Furthermore, the inappropriate use of amoxicillin (e.g. in children affected by an Epstein–Barr virus infection) can lead to unpleasant rash conditions [17]. Future studies should address the frequency of the mentioned side effect in children with pharyngitis inappropriately managed with antibiotics in emergency units. Nearly 50% of the emergency units administered amoxicillin for a duration of less than 10 days, which is the presently advised course of treatment [4]. While a treatment duration shorter than 10 days is administered to adults, its effectiveness in pediatric patients is less established and requires further investigation [18]. Taken together, these data indicate that the treatment of pharyngitis in the majority of children seen at emergency units is not consistent with international recommendations. These findings have significant implications in light of the growing issue of antimicrobial resistance on a global scale. Italy ranks among the European nations with the highest prevalence of antibiotic prescribing, including the use of broad-spectrum antibiotics for both adult and pediatric populations [19]. It has been observed that Italian general practitioners tend to overprescribe antibiotics for pediatric pharyngitis, with less than 60% of patients confirmed by rapid antigen test receiving appropriate antibiotic therapy [20]. In contrast, a recent study conducted in the United States found that while only one-third of pharyngitis cases in an emergency room were attributed to Group A β-hemolytic streptococcus, more than half of the cases were treated with antibiotics [21]. One possible explanation for the overuse of antibiotics could be the fear of rheumatic fever, which remains a major complication of untreated Group A β-hemolytic streptococcus pharyngitis in many countries, including Italy [22]. To address the issue of antimicrobial resistance, local and international guidelines should emphasize the use of clinical scores in combination with rapid antigen tests before initiating treatment, and sensitize emergency unit physicians to the appropriate use of antibiotics. This approach would promote correct antimicrobial stewardship while protecting patients from the potential complications of untreated Group A β-hemolytic streptococcus pharyngitis.

This study has at least three limitations that should be considered. Firstly, the questionnaire was not validated and only pilot tested together with an inter-rate reliability evaluation. Secondly, the responses may not fully reflect everyday clinical practice in the emergency units. Studies in the future should retrieve data from patient medical records to validate the results of this study. Third, it was limited to emergency units and did not consider primary care physicians, who take care of several cases of pharyngitis in Italy [20].

Despite these limitations, the study has several strengths. Firstly, the high response rate provides confidence in the representativeness of the sample. Secondly, the survey captured a wide range of emergency unit profiles, from small to large units, which contributes to a more accurate representation of pharyngitis management practices. Finally, the survey covered multiple aspects of the clinical approach to pediatric pharyngitis, providing a comprehensive overview of current practices.

## Conclusion

The current study shows a mismanagement of pharyngitis in a considerable number of emergency units. This might result in inappropriate antibiotic prescription or even in the development of complications for children. Nevertheless, the provision of rapid antigen tests in all hospitals could enhance the diagnostic and therapeutic approach to pharyngitis. In the context of the increase in antimicrobial resistance worldwide, it is crucial for emergency unit physicians to prioritize the appropriate management of pharyngitis to improve patient outcomes and minimize negative consequences associated with antibiotic overuse.

### Supplementary Information

**Additional file 1. **

## Data Availability

Data and materials are available upon reasonable request to the corresponding author.
