# Supplementary material for: Nationwide survey on the management of pediatric pharyngitis in Italian emergency units
Source: Ital J Pediatr. 2023 Sep 5;49:114. doi: 10.1186/s13052-023-01514-8 (PMC10481466; doi:10.1186/s13052-023-01514-8)
Supplement: Supplementary file 1 — Additional file 1. [file 13052_2023_1514_MOESM1_ESM.docx]

**Nationwide Survey on the Management of Pediatric Pharyngitis in Italian Emergency Units**

**SUPPLEMENTARY MATERIAL**

**SURVEY**

1. How many children are visited in your emergency unit annually?

- 5.000
- 5.000-10.000
- 10.000-20.000
- >20.000

**Result**

28 (26%) 5,000 cases; 26 (24%) between 5,000 - 10,000 cases; 41 (38%) between 10,000 - 20,000 cases; 12 (11%) 20,000 cases

1. How many cases of pharyngitis are managed in your emergency unit annually?

- <100
- 100-500
- 500-1000
- >1000

**Result**

9 (8%) <100 cases; 42 (39%) between 100 - 500 cases; 29 (27%) between 500 - 1000 cases; 27 (25%) >1000 cases

1. Are clinical scores used for the diagnosis of Group A β-hemolytic streptococcus pharyngitis in your emergency unit?
   - Yes, always
   - Yes, more than 50% of cases
   - Yes, less than 50% of cases
   - No, never

**Result**

10 (9%)Yes, always, 18 (16%) Yes, more than 50% of cases, 25 (23%) Yes, less than 50% of cases, 54 (50%) No, never

1. What score is mostly used?
   - Centor
   - McIsaac
   - Breese

**Result**

42 (80%) McIsaac, 6 (11%) Centor, 4 (8%) Breese

1. Does your hospital provide your emergency unit with a rapid antigen test to diagnose Group A β-hemolytic streptococcus pharyngitis?
   - Yes
   - No

**Result**

42 (43%) Yes, 60 (56%) No

1. If a child presents with pharyngitis, how often is a rapid antigen test used?
   - Always
   - Depends on the individual doctor
   - Based on the clinical scores
   - Never

**Result**

5 (10%) Always, 32 (68%) Depends on the individual doctor, 9 (19%) Based on the clinical scores, 1 (2%) Never

1. Which of the following areas is sampled to identify Group A β-hemolytic streptococcus with a rapid antigen test?
   - Posterior pharyngeal wall
   - Posterior pharyngeal wall and both tonsils
   - Both tonsils
   - Oral cavity, tonsils and the posterior pharyngeal wall
   - Only the oral cavity

**Result**

3(6%) Posterior pharyngeal wall, 28 (59%) Posterior pharyngeal wall and both tonsils, 12(25%) Both tonsils, 4 (8%) Oral cavity, tonsils and the posterior pharyngeal wall, 0 (0%) Only the oral cavity

8. What type of rapid antigen test is utilized? (open question)

**Result** (most common answers)

C**-** test (N=4, 11%) and Clear-view (N=5, 15%).

9. Are other microbiological methods used to diagnose Group A β-hemolytic streptococcus?

- - Throat culture
  - Moleculer biology tecniques (e.g. Polimerase Chain Reaction)
  - GABHS antibody titers
  - No

**Result**

45 (42%) Throat culture, 7 (6%) Moleculer biology tecniques (RT-PCR), 2 (2%) GABHS antibody titers, 53 (49%) No

10. In what cases is a throat culture performed? (open question)

**Result** (most common answer)

39(88%) At the discretion of the individual physician

11. When is an antimicrobial therapy prescribed to a child with suspected Group A β-hemolytic streptococcus pharyngitis (based on medical history and physical examination)?

- - Immediately without performing other tests
  - Only after a positive rapid antigen test
  - Only after a positive throat culture
  - In case of negative rapid antigen test and throat culture but high anti-streptolysin antibody titers

**Result**

50(47%) Immediately without performing other tests, 41(38%) Only after a positive rapid antigen test, 16 (15%) Only after a positive throat culture

12. In case of a child with suspected or confirmed Group A β-hemolytic streptococcus pharyngitis (single episode) without complications, what is the first line treatment?

- - Amoxicillin
  - Amoxicillin- clavulanate
  - Cephalosporins
  - Macrolides
  - Cotrimoxazole

**Result**

81 (76%) Amoxicillin, 26 (24%) Amoxicillin-Clavulanate

13. If cephalosporins or macrolides are used, please name the type.

**Result**

No response.

14. What is the dosage of the first-line treatment (specify in mg/kg/day)?

**Result**

75 (71%) 50mg/kg/day, 2 (2%) <50mg/kg/day, 29 (27%) >50mg/kg/day.

15. What is the duration of the first-line treatment?

**Result**

48 (45%) 10-day regimen, 56 (53%) shorter than 10 days. 2(2%) longer than 10 days.

16. In case of a child with suspected or confirmed Group A β-hemolytic streptococcus pharyngitis (single episode) without complications, what is the second-line treatment?

- - Amoxicillin
  - Amoxicillin- clavulanate
  - Cephalosporins
  - Macrolides
  - Cotrimoxazole

**Result**

56 (52%) Cephalosporins, 24 (22%) Macrolides, 22 (20%) Amoxicillin-Clavulanate, 5 (5%) Amoxicillin

17. If cephalosporins or macrolides are used, please name the type.

**Result**

17 (21%) Clarithromycin, 5 (6%) Azithromycin, 11 (14%) Cefpodoxime proxetyl, 9 (11%) cefixime, 10 (13%) Cefaclor

18. In case of a child with recurrent Group A β-hemolytic streptococcus pharyngitis (3 or more episodes in 6 months) without complications, what is the first-line treatment?

- - Amoxicillin
  - Amoxicillin- clavulanate
  - Cephalosporins
  - Macrolides
  - Cotrimoxazole

**Result**

28 (26%) Amoxicillin, 58 (54%) Amoxicillin- clavulanate, 16 (15%) Cephalosporins, 5 (5%) Macrolides, 0 (0%) Cotrimoxazole

19. If cephalosporins or macrolides are used, name the type.

**Result**

3 (3%) Clarythromycin, 1 (1%) azithromycin, 3 (3%) cefaclor, 4 (4%) cefixime, 6 (6%) cefpodoxime, 2 (2%) ceftriaxone

20. In case of a child with recurrent Group A β-hemolytic streptococcus pharyngitis (3 or more episodes in 6 months) without complications, what is the second-line treatment used in your emergency unit?

- - Amoxicillin
  - Amoxicillin- clavulanate
  - Cephalosporins
  - Macrolides
  - Cotrimoxazole

**Result**

4 (4%) Amoxicillin, 13 (12%)Amoxicillin- clavulanate, 64 (60%) Cephalosporins, 26 (24%) Macrolides, 0 (0%) Cotrimoxazole

21. If cephalosporins or macrolides are used, please name the type (open question)

**Result**

19 (18%) Clarythromycin, 7 (7%) azithromycin, 21 (20%) cefaclor, 15 (14%) cefixime, 21 (20%) cefpodoxime, 7 (7%) ceftriaxone

22. Is a microbiological follow-up (repeat rapid antigen test or throat culture) for children with GABHS pharyngitis requested in your emergency unit?

- - Yes, always
  - Yes, if there is no symptom resolution
  - Yes, only if the child has recurrent pharyngitis
  - No, never

**Result**

6(5.6%) Yes, always, 23 (21%) Yes, if there is no symptom resolution, 17 (16%) Yes, only if the child has recurrent pharyngitis, 61 (57%) Never

23. In which cases an otorhinolaryngology consult is requested in your emergency unit?

- - Fever for more than 72 hours after the start of antimicrobial treatment
  - In case of local complications
  - Never
  - I do not have an otorhinolaryngology specialists in my hospital

**Result**

5 (4%) Fever for more than 72 hours after the start of antimicrobial treatment, 99 (92%) In case of local complication, 6 (6%) Never, 0 (0%) I do not have an otorhinolaryngology specialists in my hospital

24. In which cases is an infectious diseases consult requested in your emergency unit?

- - Fever for more than 72 hours after the start of antimicrobial treatment
  - In case of local complications
  - Never
  - I do not have infectious diseases specialists in my hospital

**Result**

23 (21%) Fever for more than 72 hours after the start of antimicrobial treatment, 15 (14%) In case of local complication, 70 (65%) Never, 4 (4%) I do not have an infectious diseases specialists in my hospital

25. Does your emergency unit have internal guidelines to manage GABHS pharyngitis?

- - Yes
  - No

**Result**

78 (73%) no, 29 (27%) yes

**Name of the centers which participated the study**

| Name of the Hospital - City |  |
| --- | --- |
| \| - Ospedale S. Croce e Carle- Cuneo \| \| --- \| \| - Ospedale Maggiore della Carità- Novara \| \| - ASST Grande Ospedale Metropolitano Niguarda- Milan \| \| - IRCCS Materno Infantile Burlo Garofolo- Trieste \| \| - Ospedale Donna e Bambino AOUI- Verona \| \| - Ospedale San Raffaele- Milan \| \| - Azienda Ospedaliera Regionale "San Carlo" -Potenza \| \| - Arcispedale Santa Maria Nuova- Reggio Emilia \| \| - Ospedale San Luca- Lucca \| \| - Ospedale Filippo Del Ponte- Varese \| \| - Ospedale della Misericordia- Grosseto \| \| - Ospedale Papa Giovanni XXIII- Bergamo \| \| - IRCCS Policlinico ospedaliero -universitario di Bologna \| \| - Ospedale G. Di Maria Avola- Siracusa \| \| - Ospedale di Rieti- Rieti \| \| - Ospedale Paolo Dettori Tempio- Pausania \| \| - Ospedale Infermi- Rimini \| \| - Policlinico di Modena- Modena \| \| - Pronto Soccorso Pediatrico, Ospedale "G. Salesi" - Ancona \| \| - Ospedale "S Spirito" Pescara- Pescara \| \| - Ospedale Giovanni XXIII- Bari \| \| - Ospedale Civile di Imperia- Imperia \| \| - ASST Spedali Civili di Brescia- Brescia \| \| - Fondazione Policlinico Agostino Gemelli- Roma \| \| - AORN Sant'Anna e San Sebastiano- Caserta \| \| - Presidio Ospedaliero "Giovanni Paolo II" - Ragusa \| \| - Sant'Antonio Abate- Gallarate \| \| - Ospedale San Paolo- Milan \| \| - Ospedale di Circolo di Busto Arsizio- Busto Arsizio \| \| - Ospedale Civile di Sondrio- Sondrio \| \| - Ospedale Carlo Poma- Mantua \| \| - Ospedale Bonomo- Andria \| \| - Azienda Ospedaliera Universitaria Sassari- Sassari \| \| - Ospedale San Gerardo- Monza \| \| - Ospedale G. Fracastoro- San Bonifacio \| \| - Ospedale Generale Provinciale di Saronno- Saronno \| \| - Ospedale Provinciale di Bolzano- Bolzano \| \| - Ospedale San Bortolo di Vicenza- Vicenza \| \| - Ospedale “C. e G. Mazzoni- Ascoli Piceno \| \| - Azienda Ospedaliera Universitaria di Pisa- Pisa \| \| - Ospedale dei Bambini Azienda Ospedaliero Universitaria di Parma- Parma \| \| - Ospedale Santa Maria della Misericordia- Udine \| \| - Ospedale della Donna e del Bambino-AOUI- Verona \| \| - Ospedale San Paolo di Monfalcone- Gorizia \| \| - Azienda Ospedaliera di Padova Dipartimento A.I. per la Salute-Padova \| \| - ASST Melegnano Martesana Vizzolo Predabissi- Vizzolo Predabissi \| \| - Giovanni Paolo II- Lamezia Terme \| \| - Ospedale PIO XI - Desio \| \| - P.O. S. Antonio Abate - Trapani \| \| - IRCCS Gaslini- Genua \| \| - Presidio Ospedaliero San Francesco- Nuoro \| \| - Ospedale S. Maria del Carmine- Rovereto \| \| - Azienda Ospedaliero-Universitaria Senese- Siena \| \| - ASST OVEST Ospedale di Legnano- Legnano \| \| - ASST-Lariana Sant'Anna- Como \| \| - Ospedale S. Chiara- Trento \| \| - Ospedale Maggiore di Lodi- Lodi \| \| - Ospedale Castelli- Verbania \| \| - Ca’ Granda Ospedale Maggiore Policlinico- Milan \| \| - Ospedale dei Bambini Vittore Buzzi- Milan \| \| - Policlinico Riuniti- Foggia \| \| - Ospedale Guglielmo da Saliceto, Piacenza - Piacenza \| \| - Ospedale "Bolognini"- Seriate \| \| - Ospedale Santa Maria della Misericordia- Perugia \| \| - Ospedale Infantile Regina Margherita- Turin \| \| - Ospedale San Giuseppe Moscati di Avellino- Avellino \| \| - Ospedale Martini- Turin \| \| - Ospedale Santa Maria degli Angeli- Pordenone \| \| - Ospedale "G. Mazzini"- Teramo \| \| - Ospedale Beauregard- Aosta \| \| - Ospedale SS Giovanni e Paolo- Venezia \| \| - Ospedale Mons. Dimiccoli- Barletta \| \| - Ospedale Santa Maria della Misericordia- Rovigo \| \| - Catanzaro Azienda Pugliese- Ciaccio \| \| - Azienda Ospedaliera Universitaria Sant'Anna- Ferrara \| \| - C.T.O - Iglesias \| \| - Ospedale Cà Foncello- Treviso \| \| - ASST Ovest Milanese - Presidio Ospedaliero Magenta- Magenta \| \| - Ospedale SS. Annunziata di Chieti- Chieti \| \| - Ospedale Madonna delle Grazie - Matera \| \| - ASST Santi Carlo e Paolo- Milan \| \| - Nuovo Ospedale Prato-Santo Stefano- Prato \| \| - Città di Castello e Gubbio-Gualdo- Tadino \| \| - Ospedale San Paolo/ Santa Corona Pietra Ligure- Savona \| \| - Ospedale Generale Provinciale di Macerata- Macerata \| \| - Azienda Ospedaliera San Pio- Benevento \| \| - Ospedale Santa Maria della Misericordia di Urbino- Urbino \| \| - Ospedale Mater Salutis di Legnago- Verona \| \| - Azienda Ospedaliera Pediatrica Santobono-Pausilipon- Napoli \| \| - Ospedale San Leopoldo- Merate \| \| - Ospedale "SS Annunziata"- Taranto \| \| - Ospedale San Giovanni Battista- Foligno \| \| - Ospedale S. Matteo degli infermi- Spoleto \| \| - Fondazione IRCCS Policlinico San Matteo- Pavia \| \| - Presidio Ospdaliero Macedonio Melloni- Milan \| \| - Ospedale S. Timoteo- Termoli \| \| - Ospedale Maggiore ASST- Cremona \| \| - Ospedale San Giovanni di Dio- Agrigento \| \| - Ospedale Santa Maria della Scaletta- Imola \| \| - Ospedale Morgagni Pierantoni- Forlì \| \| - Ospedale S. Antonio Abate- Cantù \| \| - Ospedale Cardinal Massaia- Asti \| \| - Ospedale di Lavagna- Genova \| \| - Ospedale Civile di Guastalla- Reggio Emilia \| \| - Azienda Ospedaliera Universitaria di Meyer- Firenze \| \| - Ospedale Santa Maria delle Croci- Ravenna - Santa Maria delle Stelle, Melzo, Ospedale di Cernusco, Cernusco sul Naviglio \| |  |
